# Sweeping beauty: is grassland arthropod community composition effectively estimated by sweep netting?

**DOI:** 10.1002/ece3.688

**Published:** 2013-08-22

**Authors:** Ryan D Spafford, Christopher J Lortie

**Affiliations:** Department of Biology, York University4700 Keele St., Toronto, ON, M3J 1P3, Canada

**Keywords:** Bioindicator, effect size, grassland, insect, method comparison, pan trap, rarefaction, species richness, sweep net

## Abstract

Arthropods are critical ecosystem components due to their high diversity and sensitivity to perturbation. Furthermore, due to their ease of capture they are often the focus of environmental health surveys. There is much debate regarding the best sampling method to use in these surveys. Sweep netting and pan trapping are two sampling methods commonly used in agricultural arthropod surveys, but have not been contrasted in natural grassland systems at the community level. The purpose of this study was to determine whether sweep netting was effective at estimating arthropod diversity at the community level in grasslands or if supplemental pan trapping was needed. Arthropods were collected from grassland sites in Montana, USA, in the summer of 2011. The following three standardized evaluation criteria (consistency, reliability, and precision) were developed to assess the efficacy of sweep netting and pan trapping, based on analyses of variations in arthropod abundances, species richness, evenness, capture frequency, and community composition. Neither sampling method was sufficient in any criteria to be used alone for community-level arthropod surveys. On a taxa-specific basis, however, sweep netting was consistent, reliable, and precise for Thysanoptera, infrequently collected (i.e., rare) insects, and Arachnida, whereas pan trapping was consistent, reliable, and precise for Collembola and bees, which is especially significant given current threats to the latter's populations worldwide. Species-level identifications increase the detected dissimilarity between sweep netting and pan trapping. We recommend that community-level arthropod surveys use both sampling methods concurrently, at least in grasslands, but likely in most nonagricultural systems. Target surveys, such as monitoring bee communities in fragmented grassland habitat or where detailed information on behavior of the target arthropod groups is available can in some instances employ singular methods. As a general ecological principle, consistency, reliability, and precision are appropriate criteria to evaluate the applicability of a given sampling method for both community-level and taxa-specific arthropod surveys in any ecosystem.

## Introduction

Arthropods represent one of the most successful taxa on Earth with estimates for global species richness approaching 10 million (Mora et al. 2011). Arthropods shape ecosystem functioning globally in both natural and agricultural systems (Losey and Vaughan 2006; Isaacs et al. 2009). Important services include pollination (Klein et al. 2007; Ollerton et al. 2011), nutrient cycling (Seastedt and Crossley 1984; Christiansen et al. 1989; Pramanik et al. 2001; Meyer et al. 2011), and biological control of agroecosystem pests and disease vectors (Howarth 1991). As arthropods are critical components within all terrestrial ecosystems, it is important for researchers to be able to quickly, accurately, and reliably census them both across a variety of field conditions and with different end goals, for example, as insect pests in agricultural crops (Sane et al. 1999; McLeod 2000), food items for alpine birds (Norment 1987), or indicators of habitat restoration success (Bennett and Gratton 2013). However, the overall effectiveness of different arthropod sampling methods has been only cursorily explored. The majority of existing studies have contrasted sampling methods in row crops including soybean (Shepard et al. 1974; Mayse et al. 1978; Kogan and Pitre 1980; Bechinski and Pedigo 1982; Deighan et al. 1985), corn and sweet potatoes (Prasifka et al. 2007; Reed et al. 2010), peanuts (Kharboutli and Mack 1993), cotton (Garcia et al. 1982; Nuessly and Sterling 1984; Kharboutli and Allen 2000), and snap bean (McLeod 2000). Other studies have contrasted sampling methods in tropical forests (Sabu et al. 2011; Cooper et al. 2012; Lamarre et al. 2012), coastal sage scrub (Buffington and Redak 1998), northern tundra (Norment 1987), shrub/mixed grass prairie (Doxon et al. 2011), and experimental fields (Evans and Bailey 1993; Roulston et al. 2007). Typically, these method contrasts are done in tandem to ensure that direct comparisons can be made, but this has not been examined in depth in natural grassland systems. A summary of these contrasts is provided in Table 1. The implication of arthropod sampling in grasslands is important in general because grasslands account for nearly 41% of the Earth's terrestrial surface cover (White et al. 2000). Humans also dramatically impact these systems through urban development, agricultural processes, and introductions of invasive plants. Arthropods are thus potentially important indicators of ecosystem health and function and effective sampling knowledge is critical.

**Table 1:** Summary of existing arthropod sampling method contrasts

Habitat type	Article of reference	Methods evaluated	Recommendation
Agricultural	Shepard et al. (1974)	Sweep net, vacuum, ground cloth	Methods were taxa specific. No single method was best overall.
	Mayse et al. (1978)	Sweep net, direct observation, clam trap	Direct observation is the best overall sampling method.
	Kogan and Pitre (1980)	Direct observation, ground cloth, sweep net, vacuum	Could not access article.
	Bechinski and Pedigo (1982)	Sweep net, plant shake, vacuum net	Plant shake is the best overall sampling method.
	Garcia et al. (1982)	Direct observation, modified Berlese funnel, whole plant collection	Combination of Berlese funnel and whole plant collection recommended.
	Nuessly and Sterling (1984)	Vacuum, modified drop cloth	Vacuum sampling best overall sampling method.
	Deighan et al. (1985)	Sweep net, ground cloth, direct observation	Methods were taxa specific. No single method was best overall.
	Kharboutli and Mack (1993)	Beat sheet, pitfall trap, sweep net	Methods were taxa specific. No single method was best overall.
	Kharboutli and Allen (2000)	Beat sheet, sweep net, blower	Methods were taxa specific. No single method was best overall.
	McLeod (2000)	Cage aerosol, sweep net, drop cloth	Methods were taxa specific. No single method was best overall.
	Prasifka et al. (2007)	Pitfall trap, litter bag	Methods were taxa specific. No single method was best overall.
	Reed et al. (2010)	Sweep net, hand vacuum, leaf blower	Sweep netting is the best overall sampling method.
Tropical forest	Sabu et al. (2011)	Pitfall trap, Winkler extractor, Berlese funnel	Methods were taxa specific. No single method was best overall.
	Cooper et al. (2012)	Branch clipping, sweep netting	Sweep netting is the best overall sampling method.
	Lamarre et al. (2012)	Windowpane trap, malaise trap	Methods were taxa specific. No single method was best overall.
Coastal sage scrub	Buffington and Redak (1998)	Vacuum, sweep net	Vacuum sampling is the best overall sampling method.
Northern tundra	Norment (1987)	Sticky board, pitfall trap, sweep net	Methods were taxa specific. No single method was best overall.
Shrub/mixed grass prairie	Doxon et al. (2011)	Vacuum, sweep net	Methods were taxa specific. No single method was best overall.
Experimental fields	Evans and Bailey (1993)	Pan trap, sweep net	Methods were taxa specific. No single method was best overall.
	Roulston et al. (2007)	Pan trap, sweep net	Methods were taxa specific. No single method was best overall.

Sweep netting and pan trapping are two common methods used to sample arthropods associated with low-lying flowering vegetation in a wide variety of habitat types including grasslands (Roulston et al. 2007; Yi et al. 2012). Although sweep netting can be labor intensive, it is a powerful tool for quickly sampling a wide range of arthropod taxa in a short period of time (Yi et al. 2012). Sweep netting is considered a passive sampling method (i.e., no chemical, visual, or form lure is used to attract arthropods) without a bias toward the population density and trapping susceptibility of target arthropods (Melbourne 1999; Mazon and Bordera 2008; Yi et al. 2012). Conversely, pan trapping is an active sampling method. The colored bowls mimic flowers and are effective at capturing many species of bees, particularly Halictidae, but also Lepidoptera, flower-visiting flies (Roulston et al. 2007), leafhoppers, and other Hemiptera (Rodriguez-Saona et al. 2012). Furthermore, bowl color influences the quality and magnitude of pan trap catches (Vrdoljak and Samways 2012), wherein white and yellow colored bowls are particularly attractive to many species of Diptera and Hymenoptera (Disney et al. 1982; Mazon and Bordera 2008; Vrdoljak and Samways 2012) and blue colored bowls are attractive to Stephanid wasps and female members of the bee species *Andrena lamnanthis* (Aguiar and Sharkov 1997; Leong and Thorp 1999). Pan trapping is thus inexpensive, but it is also very sensitive to environmental conditions including rainfall and high winds (Yi et al. 2012), and is also biased toward capturing specific arthropod taxa (Nuttman et al. 2011; Saunders and Luck 2013). Conversely, sweep netting is robust and broad in terms of arthropod taxa capture (Orlofske et al. 2010), but requires more human effort and an experienced sampler. These two methods are both appropriate for grassland arthropods, but to date, they have not been contrasted in parallel at the community level in a natural grassland system.

Here, we present a parallel contrast of sweep netting and pan trapping in a natural grassland system to determine whether either method is an adequate standalone sampling method based on the following three criteria: consistency, reliability, and precision. To evaluate consistency (i.e., the capacity to detect true patterns), mean seasonal arthropod abundance, morphospecies richness, and morphospecies evenness were compared between sweep netting and pan trapping through the use of effect size estimates and meta-analyses. Reliability (i.e., the variation in repeated measurements) was evaluated through chi-squared tests of seasonal frequencies of arthropod capture between sweep netting and pan trapping. Finally, precision (i.e., the spatial precision in repeated measurements) for each method was evaluated through comparisons of sweep net and pan trap dispersion coefficients for mean seasonal abundances of major arthropod groups as well as through the examination of spatial aggregations of morphospecies compositions within an nonmetric multidimensional scaling (NMDS) ordination. A standalone arthropod sampling method that is consistent, reliable, and precise for all arthropod groups would be ideal as it would permit conservation biologists and land managers to not only accurately quantify the effects of natural and anthropogenic disturbances but also the success of restoration efforts in a labor and cost-effective manner at least for specific orders.

## Methods

### Study sites and arthropod sampling

Arthropods were sampled within the Blackfoot-Clearwater Wildlife Management Area in Missoula–Powell Counties, MT (47°2.966'N, 113°21.359'W). Sampling sites were characterized as intermountain grassland habitat primarily consisting of mixed grasses and forbs (bluebunch wheatgrass (*Pseudoroegneria spicata* Pursh), fescue (*Festuca* sp.), various species of Poaceae, lupine (*Lupinus spp*.), sticky geranium (*Geranium viscosissimum* Fisch. and C. A. Mey.), yarrow (*Achillea millefolium* L.), thin-leaved owl's clover (*Orthocarpus tenuifolius* Pursh [Benth.]), houndstongue hawkweed (*Hieracium cynoglossoides* Arv.-Touv.), arrowleaf balsamroot (*Balsamorhiza sagittata* Pursh [Nutt.]), and spotted knapweed (*Centaurea stoebe* L. ssp. *micranthos* [Gugler] Hayek). A total of four sites separated by at least 500 m were sampled.

**Figure 1:**
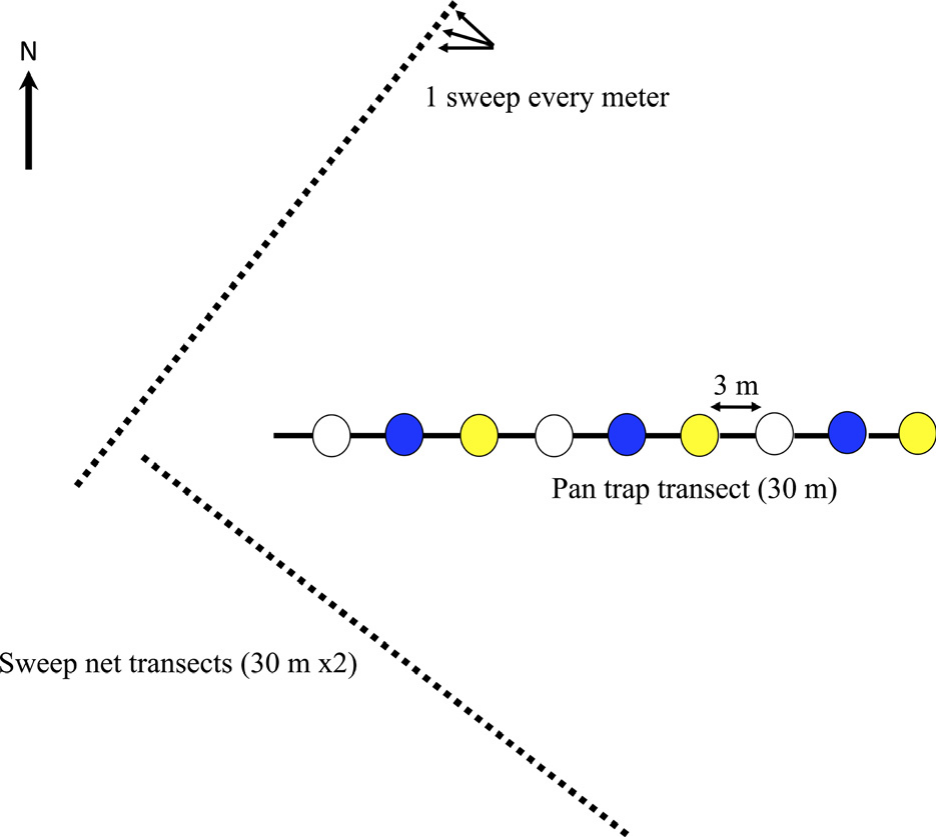
Schematic of arthropod sampling methodology (not to scale). Each transect was 30 m in length.

Permanent 30 m linear transects were established for both sweep net and pan trap arthropod sampling at each site (Fig. 1). Sweep net transects were walked slowly and one sweep was taken every meter for a total of 30 sweeps/transect with two sweep net transects established at each site. The vegetative and flowering portions of plants along each transect were swept. A single pan trap transect was also established at each site in an east–west orientation and consisted of either a white bowl (16 cm diameter), blue bowl (18.5 cm diameter), or yellow bowl (18.5 cm diameter) half filled with soapy water prepared with unscented dish detergent (NSERC-CANPOLIN 2009). Alternate colored pan traps were arranged at 3 m intervals such that nine traps were set along each 30 m transect (Fig. 1). Pan traps were placed on the ground surface (but not within dense vegetation) before 10 am, and collected after 24 h. Small differences in bowl size and the length of trap deployment (8 h vs. 24 h) have not been shown to significantly impact pan trap abundances or capture rates (Droege 2005). Arthropod catches from all pan trap colors were compiled and analyzed as recommended, thereby avoiding bias by arthropod color preferences (Toler et al. 2005). Arthropods were stored in vials of 95% ethanol until processing. At each site, arthropods were collected biweekly from early June until mid-August 2011 for a total of six sampling events. Each sweep net transect was treated as an independent sample and every color from each pan trap transect was also treated independently. Therefore, there were *N* = 48 sweep net samples and *N* = 71 pan trap samples (one pan trap sample from June 4 was lost).

Arthropods were sorted into 11 major taxonomic groups including the following: beetles (Coleoptera), flies (Diptera), true bugs (Hemiptera), ants and wasps (Hymenoptera), bees (Hymenoptera), butterflies and moths (Lepidoptera), grasshoppers, crickets, katydids (Orthoptera), thrips (Thysanoptera), spiders, mites and ticks (Arachnida), springtails (Collembola), and a larger grouping of uncommonly collected insects (i.e., rare, less than 10 instances). Bees were analyzed separately from other Hymenoptera because studies have shown them to be particularly sensitive to sampling methods such as pan trapping (Roulston et al. 2007). For species richness estimates, the major arthropod taxonomic groups were further sorted into families or higher taxa (i.e., species or morphospecies) using dichotomous keys (e.g., Borror et al. 1989; Goulet and Huber 1993; Arnett et al. 2002; Marshall 2006). Morphotyping is viewed as a reasonable surrogate for species-level identifications of difficult specimens (Oliver and Beattie 1993, 1996).

### Statistical analyses

Variation in morphospecies captures between sweep netting and pan trapping was compared using rarefaction curves generated with EstimateS v8.20 using the Mao Tau estimator (Gotelli and Colwell 2001; Colwell 2006). Rarefaction curves provide an estimate of the number of species expected for a given number of samples collected (Gotelli and Colwell 2001).

Log response ratios (LRR) were chosen as effect size estimate for arthropod abundance, morphospecies richness, and morphospecies evenness to evaluate the general consistency of sweep netting and pan trapping (Hedges et al. 1999). Sweep netting was coded as the control and pan trapping as the treatment, as sweeps are a more commonly used arthropod sampling method. Therefore, positive mean values significantly different from 0 indicate that pan trapping increases the effectiveness of sweeps and is needed, whereas mean estimates that do not significantly differ from 0 indicate that pan trapping supplementation does not differ from sweep netting only (Hedges et al. 1999). Zero values in raw data are ecologically relevant, but do not allow for the calculation of LRR. To address this issue, we added 1 to all observations of abundance and richness, and 0.01 to evenness (which only spans between 0 and 1) before calculating effect size estimates. Three separate meta-analyses for arthropod abundance, morphospecies richness, and morphospecies evenness by major arthropod group were used to evaluate the consistency of sweep netting with sampling location as replicates. Alpha was set at *P* < 0.05, and bias-corrected confidence intervals (CI) were estimated using bootstrap procedures (Adams et al. 1997) via 9999 iterations in MetaWin 2.0 (Rosenberg et al. 2000). Heterogeneity was examined using *Q*-statistics (Hedges and Olkin 1985).

Reliability was examined by chi-squares to test for differences in the relative frequency of capture of major groups of arthropods over the entire sampling season using JMP 10 (JMP 1989-2012). Each sample was categorized based on the capture of either one or more, or greater than 10 individuals (after Prasifka et al. 2007).

The coefficient of variation (CV; σ/μ) was used to estimate the precision of each method (Zar 1974) via seasonal arthropod abundances within each major arthropod group. A lower CV suggests that a method has less variation relative to the mean (i.e., less noise) and therefore may have greater potential to detect treatment effects (Zar 1974). We natural-log transformed raw CV values to generate normal distributions confirmed via goodness-of-fit tests as *P* > 0.05 (fail to reject *H*_0_ for normality). A generalized linear model (GLM) with post hoc comparisons using 95% CI for the distribution of differences was then done between sweep netting and pan trapping for each arthropod group. NMDS was used to compare arthropod morphospecies specificity within and between sampling methods (McCune and Grace 2002). The stability of the solution was assessed by plotting stress versus iteration number with a stability criterion of 0.00001 (McCune and Grace 2002). Monte Carlo permutations were used to assess the probability that a similar final stress could have occurred by chance for each dimension. Pearson's r2 was used to correlate distance in the ordination space with distance in the original space to describe the proportion of variance explained by each axis. Multiresponse permutation procedures (MRPP) were then used to test for differences in arthropod morphospecies assemblages between sweep netting and pan trapping (PC-ORD version 5.0; McCune and Mefford 1999) by generating an overall probability that community assemblage is less dissimilar within groups than between groups (McCune and Grace 2002). Average within group dissimilarity was estimated using the Sørensen (Bray–Curtis) distance measure because it is well suited to the variability inherent in community-scale data sets (McCune and Grace 2002). Significant effects for all analyses were considered at the alpha level of *P* < 0.05.

## Results

**Figure 2:**
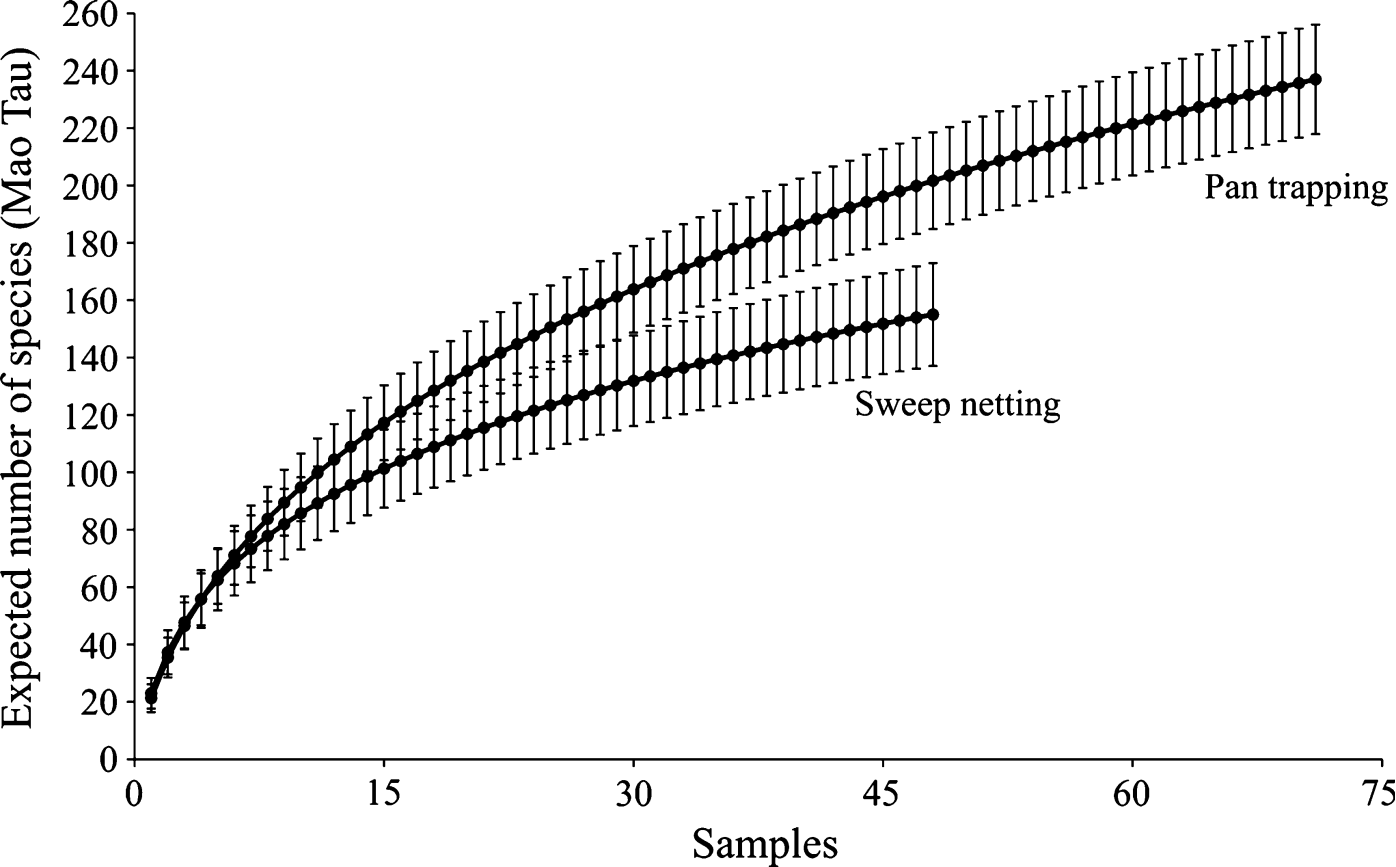
Sample-based rarefaction curves plus 95% CI for arthropod communities sampled using sweep netting and pan trapping at four sites in the Blackfoot-Clearwater Wildlife Management Area, MT.

A total of 6397 arthropods representing 155 morphospecies were collected via sweep netting, and 12,344 arthropods representing 237 morphospecies were collected via pan trapping. Given equivalent sampling effort, observed morphospecies richness was increased by nearly 50 morphospecies (nonoverlapping 95% CI) for pan trapping relative to sweep netting (Fig. 2). Rarefaction curves did not reach asymptote for either method indicating that rare arthropod species had yet to be sampled.

**Figure 3:**
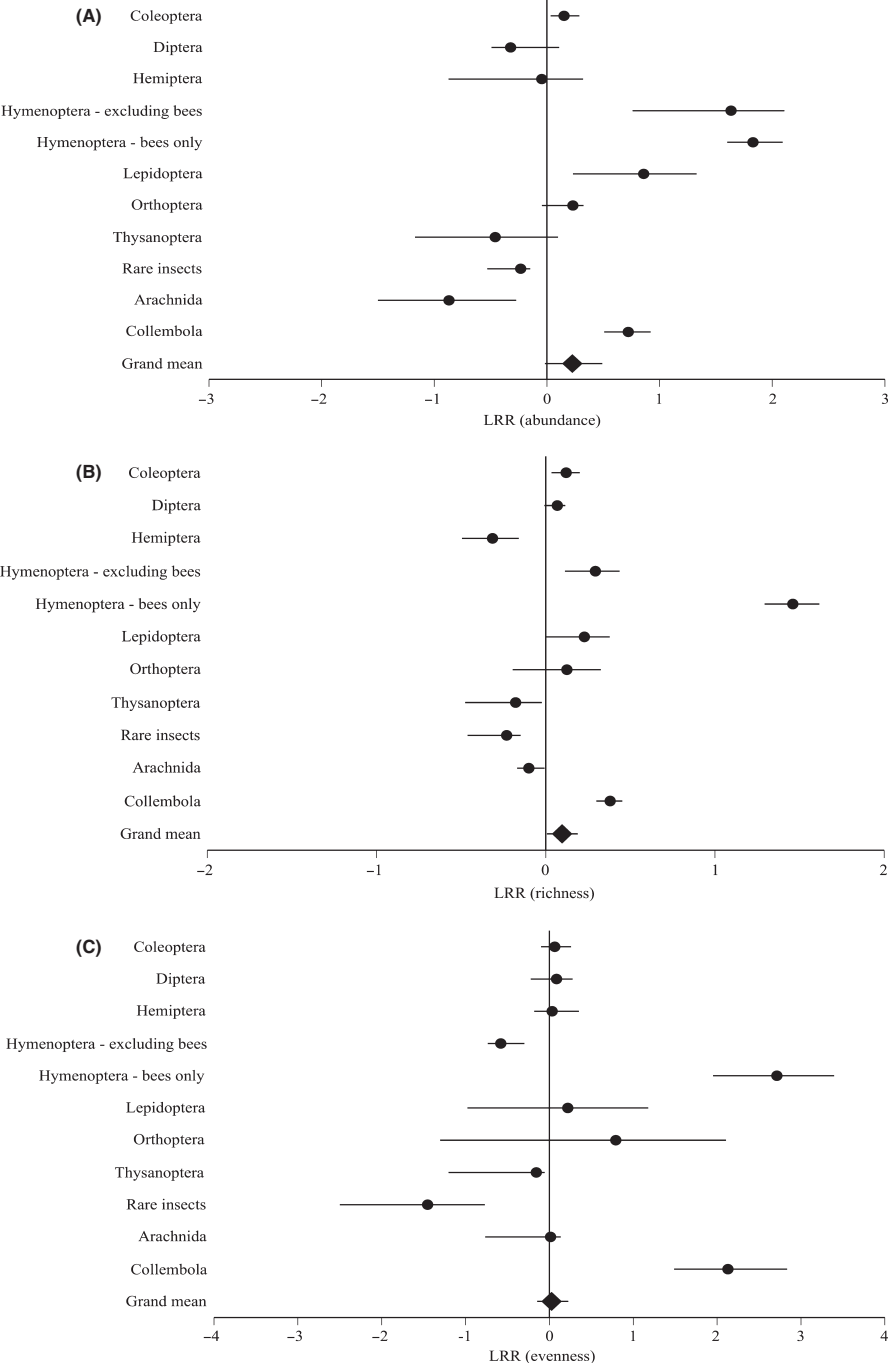
Log response ratios (±95% bootstrap confidence intervals) for mean abundance (A), morphospecies richness (B), and morphospecies evenness (C) of the major arthropod groups as measured by pan trapping (positive log response ratios [LRR]) and sweep netting (negative LRR).

There was no significant heterogeneity between or within groups in all meta-analyses of arthropod measures (abundance, richness, and evenness) (*Q*-statistics, all *P* > 0.05). The between-group heterogeneity of arthropod abundances was, however, significantly different (*Q* = 31.35; *P* = 0.00051). Arthropod species richness was significantly enhanced by pan trapping in addition to sweep netting (Fig. 3A–C, i.e., the grand mean for arthropod species richness was positive and significantly differed from no effect). At the subgroup level, abundance was enhanced by pan trapping for 45% of the major arthropod groups (Fig. 3A), morphospecies richness for 36% of the major arthropod groups (Fig. 3B), and morphospecies evenness for only 18% of the major arthropod groups (Fig. 3C). All measures were positive and significant for bee members of the order Hymenoptera and Collembola (Fig. 3). Pan trapping was not an effective addition to sweep net sampling (i.e., negative LRR values) for Arachnida, rare insects, or Thysanoptera (Fig. 3).

Reliability differed between the two methods (Table 2). Overall, sweep netting more frequently captured one or more individuals of Arachnida, Thysanoptera, and rare insects, whereas pan trapping more frequently captured one or more individuals of Collembola and bee members of the order Hymenoptera (Table 2). For samples containing greater than 10 individuals, sweep netting was more reliable for captures of Hemiptera and Thysanoptera, and pan trapping was more reliable for capturing bee members of the order Hymenoptera. All other arthropod groups showed no significant differences between methods (Table 2), and the capture frequencies of Orthoptera were low regardless of sampling method (<20%; Table 2).

**Table 2:** Chi-square test for major arthropod groups collected via sweep netting and pan trapping in intermountain grasslands

Capture frequency	Arthropod group	Frequency of collection (%)^1^		Chi-square test^2^	
	
		Sweep netting	Pan trapping	*χ*^2^	*P*-value
One or more individuals	Coleoptera	88	73	3.510	0.0610
	Diptera	100	100	–3	1.000
	Hemiptera	100	94	2.798	0.0944
	Hymenoptera – excluding bees	100	100	–	1.000
	Hymenoptera – bees only	21	77	37.056	**<0.0001**
	Lepidoptera	52	63	1.509	0.2193
	Orthoptera	13	15	0.210	0.6427
	Thysanoptera	83	56	9.473	**0.0021**
	Rare insects	15	0	11.001	**0.0009**
	Arachnida	94	65	13.350	**0.0003**
	Collembola	4	32	13.751	**0.0002**
>10 individuals	Coleoptera	29	15	3.227	0.0724
	Diptera	85	74	1.917	0.1662
	Hemiptera	88	66	6.824	**0.0090**
	Hymenoptera – excluding bees	81	89	1.350	0.2453
	Hymenoptera – bees only	0	10	5.258	**0.0218**
	Lepidoptera	0	4	2.111	0.1463
	Orthoptera	0	0	–	–
	Thysanoptera	15	1	8.137	**0.0043**
	Rare insects	0	0	–	–
	Arachnida	8	1	3.412	0.0647
	Collembola	0	0	–	–

Significance at α = 0.05 is indicated in bold font.

1 Percentage of traps from 48 sweep or 71 pan samples.

2 Chi-square test, 1 df.

3 Chi-square statistic could not be calculated.

**Figure 4:**
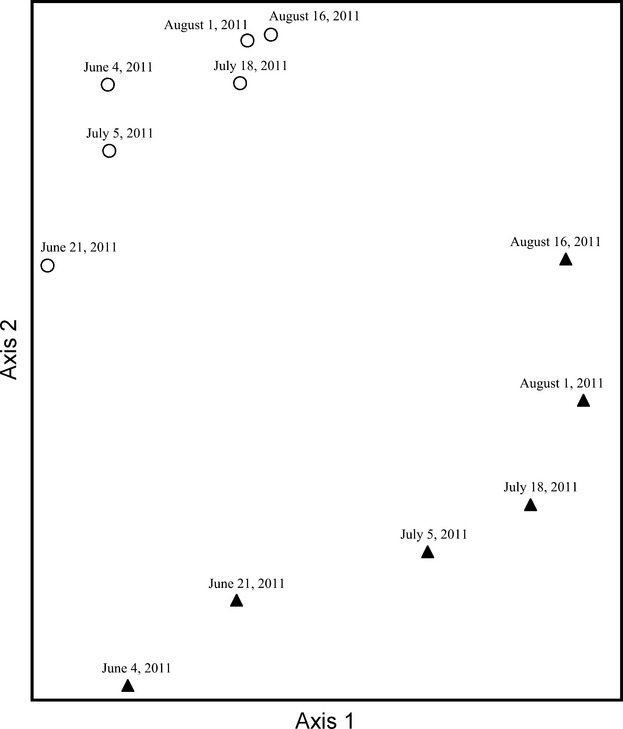
Nonmetric multidimensional scaling (NMDS) ordination of morphospecies composition from sweep netting (dark triangles) and pan trapping (open circles) over six sampling periods in 2011.

There was on average no significant difference between the patterns of CV for the two methods tested indicating broad similarity in precision (χ = 0.11, *P* = 0.82, df = 1). However, the CV associated with orders was significantly different (χ = 31, *P* = 0.0006, df = 10), and paired post hoc differences at *P* < 0.05 were found for Coleoptera, Arachnida, and Collembola (Table 3, bolded text). An NMDS ordination of arthropod morphospecies assemblages yielded a two-dimensional solution that explained 90% of the variation with a final model stress of 6.8, and a final instability of <0.00001 (Fig. 4). There was significant separation in ordination space between sampling methods with no points overlapping (MRPP, *T* = −5.9, *A* = 0.19, *P* = 0.0004). Arthropod assemblages captured via pan trapping were less dissimilar over time than those captured via sweep netting (Sørensen dissimilarity estimate of 0.656 for sweep netting and 0.487 for pan trapping).

**Table 3:** Coefficients of variation for mean seasonal abundances of the major arthropod groups collected via sweep netting and pan trapping

Arthropod group	Coefficient of variation (100 × σ/μ)	

	Sweep netting	Pan trapping
Coleoptera	**122.94**	**297.45**
Diptera	98.07	81.63
Hemiptera	82.83	115.78
Hymenoptera – excluding bees	71.10	110.92
Hymenoptera – bees only	223.20	180.48
Lepidoptera	149.46	129.86
Orthoptera	282.54	313.43
Thysanoptera	261.63	166.85
Rare insects	100.00	–
Arachnida	**83.63**	**140.89**
Collembola	**484.65**	**192.13**

Significant differences between methods at α = 0.05 is indicated with bold font. –, no individuals collected.

## Discussion

Arthropods both drive and respond to change in most ecosystems including grasslands. This study represents a first effort to contrast community-level methods for arthropod sampling in naturalized grasslands. Several contrasts of other sampling methods (e.g., sweep netting, vacuum sampling, drop cloths, pan trapping, pitfall trapping, litterbags, flight intercept traps, etc.) have been done in agricultural settings (Shepard et al. 1974; Mayse et al. 1978; Kogan and Pitre 1980; Bechinski and Pedigo 1982; Garcia et al. 1982; Nuessly and Sterling 1984; Deighan et al. 1985; Kharboutli and Mack 1993; Kharboutli and Allen 2000; McLeod 2000; Prasifka et al. 2007; Reed et al. 2010), tropical forests (Sabu et al. 2011; Cooper et al. 2012; Lamarre et al. 2012), coastal sage scrub (Buffington and Redak 1998), northern tundra (Norment 1987), shrub/mixed grass prairie (Doxon et al. 2011), and experimental fields (Evans and Bailey 1993; Roulston et al. 2007), but none in grasslands. The consensus from these general contrasts, however, is that an individual sampling method may be appropriate for specific arthropod taxa, but community-level surveys require the use of more than one method to capture adequate estimates of arthropod abundance and richness. The contrasts herein support this consensus and suggest that neither sweep netting nor pan trapping should be used alone for community-level arthropod surveys in grassland systems for the majority of arthropod taxa. If rapid assessment is needed for certain taxa, such as Collembola and bee members of the order Hymenoptera, pan trapping in grasslands was shown to be consistent, reliable, and precise. Sweep netting was consistent, reliable, and precise for Thysanoptera, infrequently collected insects, and Arachnida if required. The purpose and scope of a given study can therefore determine whether both methods are needed. Clearly, for community-level estimates, however, sweep netting and pan trapping together are needed to provide a more robust estimate of diversity.

Use of more than one method is particularly critical because most field-based sampling regimes are also likely not intensive enough to document more than a minor subset of any true arthropod population. This criticism of course applies to all sampling in ecological systems, that is, we sample as intensively as possible but never sample every individual, but arthropods are highly mobile and often difficult to capture/recapture (Kiss and Samu 2000). Consequently, addition of supplemental techniques that introduce limited researcher collection time and that also sample different suites of species are an excellent proxy for generally increasing sampling intensity. Importantly, these findings show that allocation of additional effort to sweeps would still not enhance the effective capture rates of bees. However, given that the vast majority of studies generally use only sweeps to estimate the “true” community, we propose that it is appropriate to examine changes in the effect size estimates associated with capture rates when an additional method (i.e. treatment) is added. This is the simplest way to examine the criteria we proposed herein, and whilst it may not necessarily capture the “true” community, this study shows that it can be a significant step in providing broader community estimates.

Interestingly, captures of Orthoptera (primarily Acridid grasshoppers) were underrepresented by both sweep netting and pan trapping in this study. The ineffectiveness of either method to capture this group contradicts previous studies in shrub/mixed grass prairie where abundances and capture frequencies were high and reliable (Evans and Bailey 1993; Siemann et al. 1999; Doxon et al. 2011). The findings here may be due to the strong jumping/flying capabilities of this group and the susceptibility of certain species to being flushed from disturbed vegetation during daytime sampling (Larson et al. 1999). Given that our sweep netting protocol necessitated close contact with vegetative and flowering plant structures at all times and adopted a fairly low sweep trajectory, strong jumping species may have been able to elude collection (Larson et al. 1999). Similarly, the relatively small and shallow nature of our pan traps may not have effectively captured larger grasshoppers. Larger diameter pan traps (>25 cm; Evans and Bailey 1993) and increased sampling effort (i.e., more traps, a greater number of sweep net passes, and longer sampling windows) have been shown to adequately sample Orthoptera populations (Larson et al. 1999; Fielding 2011). As this group is an important food item for higher order consumers (e.g., birds, Norment 1987), a modified pan trap regime would be necessary for studies when accurate detection of abundances and species richness is crucial.

Sweep netting was more effective than pan trapping for the capture of two major arthropod taxa. Sweep netting resulted in higher abundance estimates, species richness counts, and frequencies of capture for Arachnida (spiders, harvestmen, ticks, and mites) and Thysanoptera. These arthropods use vegetative structures directly as food, shelter, and anchors for web building (Warui et al. 2005; McDonald 2007; Pearson 2009), and cling tightly to vegetation during disturbance. The vigorous action of sweep netting was therefore more effective than pan trapping (little to no vegetative disturbance) at dislodging these groups from vegetation (Parajulee et al. 2006). Furthermore, maintaining the visibility of pan traps necessitated placement outside of structurally dense vegetation patches favored by these groups (McDonald 2007). Enhanced consistency, reliability, and precision of pan trapping for Arachnida would likely be seen for ground-dwelling or wandering spiders (Gnaphosidae, Lycosidae), and in instances where nectar- and pollen-feeding Thysanoptera are visually attracted to pans (Annand 1926; Terry 2001). However, in studies focusing on Arachnida and/or Thysanoptera, sweep netting would be an adequate standalone sampling method.

Pan trapping rather than sweep netting was particularly well suited to the capture of wild bees relative to other arthropod groups. This is a critical finding given their sensitivity to habitat fragmentation (Hinners et al. 2012) and a pressing need to monitor their populations as they unfortunately experience dramatic global declines (Lebuhn et al. 2013). Insect pollinators, including wild bees, service crops to the order of 190.5 billion dollars per year (Lebuhn et al. 2013). Roulston et al. (2007) reported greater bee capture via netting than by pan trapping. However, the sweep netting protocol herein was indiscriminate on both flowering and nonflowering vegetation, whereas their protocol targeted common flowering species at their study plots (Roulston et al. 2007). Lebuhn et al. (2013) suggest that bee populations are adequately monitored regionally, nationally, and globally with pan traps alone, consistent with the results obtained herein. The specificity of pan trapping compared to sweep netting as demonstrated by the tight clustering of the NMDS ordination is likely due to this method attracting specific orders of arthropods via color (Rodriguez-Saona et al. 2012). Furthermore, the highly distinct separation in ordination space between sweep net and pan trap samples suggests that these methods collect distinct arthropod fauna at the morphospecies level, but at coarser resolutions (i.e., major arthropod groups or orders) these differences were not completely visible. Given that agricultural systems heavily rely on bees as pollinators and because grasslands are important and irreplaceable habitat for this group, a standalone sampling method to monitor fluctuations in their populations is extremely valuable. This contrast suggests that pan trapping is a convenient, and unanimously consistent, reliable, and precise method to monitor bee communities in both pristine and fragmented grassland systems.

Consistency, reliability, and precision were novel and successful criteria as a means to contrast arthropod sampling methods. We propose that their applicability likely extends to contrasts of other sampling method perhaps in most ecosystems. Furthermore, these criteria allowed us to identify sampling method strengths and deficiencies on an arthropod taxa-specific basis while also incorporating community-level arthropod abundance, species richness, and species evenness as factors. Existing contrasts have made use of criteria, notably precision (Sane et al. 1999; Kharboutli and Allen 2000; Prasifka et al. 2007; Cooper et al. 2012), but studies evaluating method performance based on a standardized set of criteria remain scarce (but see Cooper et al. 2012). Therefore, future methods contrasts could benefit from the use of the standardized criteria detailed in this study when optimization of estimates of abundance, richness, evenness, or all three factors is desired.

## Conclusions

Sweep netting and pan trapping have benefits and drawbacks in terms of their ease of use and as shown by the three evaluation criteria here their efficacy in capturing target arthropod fauna such as wild bees. Based on these criteria, we recommend that sweep netting and pan trapping be used concurrently for community-level arthropod surveys in grassland systems. Comprehensive sampling regimes will maximize community estimates of arthropod abundance and species richness (e.g., sweep netting in addition to pan trapping), and ultimately increase the accuracy of detection of treatment effects on whole arthropod communities. Projects that are narrower in scope (e.g., monitoring bee communities in fragmented grassland habitat) with information on behavior of the target arthropod groups can in some instances employ singular methods. As a general ecological principle, consistency, reliability, and precision are valid criteria to contrast the relative applicability of a given method for both community-level and taxa-specific arthropod surveys.

## References

[b1] Adams D, Gurevitch CJ, Rosenberg MS (1997). Resampling tests for meta-analysis of ecological data. Ecology.

[b2] Aguiar A, Sharkov A (1997). Blue pan traps as a potential method for collecting Stephanidae (Hymenoptera). J. Hymenopt. Res.

[b3] Annand P (1926). Thysanoptera and the pollination of flowers. Am. Nat.

[b4] Arnett RH, Thomas MC, Skelley PE, Frank JH (2002). American beetles.

[b5] Bechinski EJ, Pedigo LP (1982). Evaluation of methods for sampling predatory arthropods in soybeans. Environ. Entomol.

[b6] Bennett A, Gratton C (2013). Floral diversity increases beneficial arthropod richness and decreases variability in arthropod community composition. Ecol. Appl.

[b7] Borror DJ, Triplehorn CA, Johnson NF (1989). An introduction to the study of insects.

[b8] Buffington ML, Redak RA (1998). A comparison of vacuum sampling versus sweep-netting for arthropod biodiversity measurements in California coastal sage scrub. J. Insect Conserv.

[b9] Christiansen T, Lockwood J, Powell J (1989). Litter decomposition by arthropods in undisturbed and intensively managed mountain brush habitats. Great Basin Nat.

[b10] Colwell RK (2006). http://purl.oclc.org/estimates.

[b11] Cooper NW, Thomas MA, Garfinkel MB, Schneider KL, Marra PP (2012). Comparing the precision, accuracy, and efficiency of branch clipping and sweep netting for sampling arthropods in two Jamaican forest types. J. Field Ornithol.

[b12] Deighan J, Mcpherson RM, Ravlin WF (1985). Comparison of sweep-net and ground-cloth sampling methods for estimating arthropod densities in different soybean cropping systems. J. Econ. Entomol.

[b13] Disney RHL, Erzinclioglu YZ, Henshaw DJ, Unwin DM, Withers P, Woods A (1982). Collecting methods and the adequacy of attempted fauna surveys, with reference to the Diptera. Field Stud.

[b14] Doxon ED, Davis CA, Fuhlendorf SD (2011). Comparison of two methods for sampling invertebrates: vacuum and sweep-net sampling. J. Field Ornithol.

[b15] Droege S (2005). The bee inventory plot.

[b16] Evans E, Bailey K (1993). Sampling grasshoppers (Orthoptera: Acrididae) in Utah grasslands: pan trapping versus sweep sampling. J. Kansas Entomol. Soc.

[b17] Fielding DJ (2011). Assessment of grasshopper abundance in cereal crops using pan traps. Int. J. Pest Manage.

[b18] Garcia A, Gonzalez D, Leigh TF (1982). Three methods for sampling arthropod numbers on California cotton. Environ. Entomol.

[b20] Gotelli NJ, Colwell RK (2001). Quantifying biodiversity: procedures and pitfalls in the measurement and comparison of species richness. Ecol. Lett.

[b21] Goulet H, Huber JF (1993). Hymenoptera of the world: an identification guide to families.

[b22] Hedges LV, Olkin I (1985). Statistical methods for meta-analysis.

[b23] Hedges LV, Gurevitch J, Curtis PS (1999). The meta-analysis of response ratios in experimental ecology. Ecology.

[b24] Hinners S, Kearns C, Wessman C (2012). Roles of scale, matrix and native habitat in supporting a diverse suburban pollinator assemblage. Ecol. Appl.

[b25] Howarth FG (1991). Environmental impacts of classical biological control. Annu. Rev. Entomol.

[b26] Isaacs R, Tuell JA, Fiedler M, Gardiner M, Landis D (2009). Maximizing arthropod-mediated ecosystem services in agricultural landscapes: the role of native plants. Front. Ecol. Environ.

[b27] JMP (1989).

[b28] Kharboutli MS, Allen CT (2000). Comparison of sampling techniques for tarnished plant bug and predaceous arthropods. AAES Spec. Rep.

[b29] Kharboutli MS, Mack TP (1993). Comparison of three methods for sampling arthropod pests and their natural enemies in peanut fields. J. Econ. Entomol.

[b30] Kiss B, Samu F (2000). Evaluation of population densities of the common wolf spider *Pardosa agrestis* (Aranaeae: Lycosidae) in Hungarian alfalfa fields using mark-recapture. Eur. J. Entomol.

[b31] Klein AM, Vaissière BE, Cane JH, Steffan-Dewenter I, Cunningham SA, Kremen C (2007). Importance of pollinators in changing landscapes for world crops. Proc. Biol. Sci.

[b32] Kogan M, Pitre HN, Kogan M, Herzog DZ (1980). General sampling methods for above-ground populations of soybean arthropods. Sampling methods in soybean entomology.

[b34] Lamarre G, Molto Q, Fine P, Baraloto C (2012). A comparison of two common flight interception traps to survey tropical arthropods. ZooKeys.

[b35] Larson DP, Neill KMO, Kemp WP (1999). Evaluation of the accuracy of sweep sampling in determining grasshopper (Orthoptera: Acrididae) community composition. J. Agric. Urban Entomol.

[b101] Lebuhn G, Droege S, Connor EF, Gemmill-Herren B, Potts SG, Minckley RL (2013). Detecting insect pollinator declines on regional and global scales. Conservation biology: the journal of the Society for Conservation Biology.

[b36] Leong JM, Thorp RW (1999). Color-coded sampling: the pan trap color preferences of oligolectic and nonoligolectic bees associated with a vernal pool plant. Ecol. Entomol.

[b37] Losey J, Vaughan M (2006). The economic value of ecological services provided by insects. Bioscience.

[b38] Marshall SA (2006). Insects: their natural history and diversity: with a photographic guide to insects of eastern North America.

[b39] Mayse MA, Kogan M, Price PW (1978). Sampling abundances of soybean arthropods: comparison of methods. J. Econ. Entomol.

[b40] Mazon M, Bordera S (2008). Effectiveness of two sampling methods used for collecting Ichneumonidae (Hymenoptera) in the Cabañeros National Park (Spain). Eur. J. Entomol.

[b41] McCune B, Grace JB (2002). Analysis of ecological communities.

[b42] McCune B, Mefford MJ (1999). PC-ORD: multivariate Analysis of Ecological Data, version 5.

[b43] McDonald B (2007). Effects of vegetation structure on foliage dwelling spider assemblages in native and non-native Oklahoma grassland habitats. Proc. Okla. Acad. Sci.

[b44] McLeod PJ (2000). Comparison of insect sampling techniques in snap bean. J. Vegetable Crop Prod.

[b45] Melbourne BA (1999). Bias in the effect of habitat structure on pitfall traps: an experimental evaluation. Aust. J. Ecol.

[b46] Meyer WM, Ostertag R, Cowie RH (2011). Macro-invertebrates accelerate litter decomposition and nutrient release in a Hawaiian rainforest. Soil Biol. Biochem.

[b47] Mora C, Tittensor DP, Adl S, Simpson AGB, Worm B (2011). How many species are there on Earth and in the ocean?. PLoS Biol.

[b48] Norment CJ (1987). A comparison of three methods for measuring arthropod abundance in tundra habitats and its implications in avian ecology. Northwest Sci.

[b49] Nuessly GS, Sterling WL (1984). Comparison of D-Vac and modified drop cloth methods for sampling arthropods in cotton. Southwest Entomol.

[b50] Nuttman CV, Otieno M, Kwapong PK, Combey R, Willmer PG, Potts SG (2011). Aerial pan-trapping: a method for assessing insect pollinators in tree canopies. J. Kansas Entomol. Soc.

[b51] Oliver I, Beattie AJ (1993). A possible method for the rapid assessment of biodiversity. Conserv. Biol.

[b52] Oliver I, Beattie AJ (1996). Invertebrate morphospecies as surrogates for species: a case study. Conserv. Biol.

[b53] Ollerton J, Winfree R, Tarrant S (2011). How many flowering plants are pollinated by animals?. Oikos.

[b54] Orlofske J, Ohnesorg W, Debinski D (2010). Potential terrestrial arthropod indicators for tallgrass prairie restoration in Iowa. Ecol. Restor.

[b55] Parajulee MN, Shrestha RB, Leser JF (2006). Sampling methods, dispersion patterns, and fixed precision sequential sampling plans for western flower thrips (Thysanoptera: Thripidae) and cotton fleahoppers (Hemiptera: Miridae) in cotton. J. Econ. Entomol.

[b56] Pearson DE (2009). Invasive plant architecture alters trophic interactions by changing predator abundance and behavior. Oecologia.

[b57] Pramanik R, Sarkar K, Joy V (2001). Efficiency of detritivore soil arthropods in mobilizing nutrients from leaf litter. Trop. Ecol.

[b58] Prasifka JR, Lopez MD, Hellmich RL, Lewis LC, Dively GP (2007). Comparison of pitfall traps and litter bags for sampling ground-dwelling arthropods. J. Appl. Entomol.

[b59] Reed J, Adams L, Abel CA (2010). Comparison of three insect sampling methods in sweetpotato foliage in Mississippi. J. Entomol. Sci.

[b60] Rodriguez-Saona CR, Byers JA, Schiffhauer D (2012). Effect of trap color and height on captures of blunt-nosed and sharp-nosed leafhoppers (Hemiptera: Cicadellidae) and non-target arthropods in cranberry bogs. Crop Prot.

[b61] Rosenberg MS, Adams DC, Gurevitch J (2000). MetaWin: statistical software for meta-analysis.

[b62] Roulston TH, Smith SA, Brewster AL (2007). A comparison of pan trap and intensive net sampling techniques for documenting a bee (Hymenoptera: Apiformes) fauna. J. Kansas Entomol. Soc.

[b63] Sabu TK, Shiju RT, Vinod K, Nithya S (2011). A comparison of the pitfall trap, Winkler extractor and Berlese funnel for sampling ground-dwelling arthropods in tropical montane cloud forests. J. Insect Sci.

[b64] Sane I, Alverson DR, Chapin JW (1999). Efficiency of conventional sampling methods for determining arthropod densities in close-row soybeans. J. Agric. Urban Entomol.

[b65] Saunders ME, Luck GW (2013). Pan trap catches of pollinator insects vary with habitat. Aust. J. Entomol.

[b66] Seastedt T, Crossley D (1984). The influence of arthropods on ecosystems. Bioscience.

[b67] Shepard M, Carner GR, Turnipseed SG (1974). A comparison of three sampling methods for arthropods in soybean. Environ. Entomol.

[b68] Siemann E, Haarstad J, Tilman D (1999). Dynamics of plant and arthropod diversity during old field succession. Ecography.

[b69] Terry I, Marullo R, Mound LA (2001). Thrips: the primeval pollinators. Thrips and Tospoviruses: Proceedings of the 7th International Symposium on Thysanoptera.

[b70] Toler TR, Evans EW, Tepedino VJ (2005). Pan-trapping for bees (Hymenoptera: Apiformes) in Utah's west desert: the importance of color diversity. Pan-Pac. Entomol.

[b71] Vrdoljak SM, Samways MJ (2012). Optimising colored pan traps to survey flower visiting insects. J. Insect Conserv.

[b72] Warui C, Villet M, Young T, Jocqué R (2005). Influence of grazing by large mammals on the spider community of a Kenyan savanna biome. J. Arachnol.

[b73] White R, Murray S, Rohweder M (2000). Pilot analysis of global ecosystems (page): grassland ecosystems.

[b74] Yi Z, Jinchao F, Dayuan X, Weiguo S, Axmacher JC (2012). A comparison of terrestrial arthropod sampling methods. J. Resour. Ecol.

[b75] Zar JH (1974). Biostatistical analysis.

